# Effectiveness of Digital Interventions That Are Available for Healthcare Professionals Who Experience Psychological Trauma: A Systematic Literature Review

**DOI:** 10.1192/bjo.2025.10124

**Published:** 2025-06-20

**Authors:** Antigoni Elisseou, Athanasios Hassoulas, Roshelle Ramkisson

**Affiliations:** ^1^Cardiff University, Cardiff, United Kingdom; ^2^Manchester University NHS Foundation Trust, Manchester, United Kingdom; ^3^University of Central Lancashire, Lancashire, United Kingdom

## Abstract

**Aims:** Historically, healthcare professionals were prone to experiencing turmoil of emotions prominent to psychological trauma due to the nature of their work. The healthcare professionals were subjected to elevated risks of psychological elements, leading to mental health implications due to the aftermath of the COVID-19 pandemic. By extension, these mental health repercussions can highly affect the patients’ care as they profoundly affect the healthcare professionals from offering the best quality of care. Different types of digitalised psychological interventions exist and seem to be making an increased trend into being added into the medical field. They are becoming increasingly popular for mental health improvements due to their cost-effectiveness, their scalability, their ability to offer greater anonymity and stigma reduction compared with traditional interventions. Randomised controlled studies (RCTs) were systematically reviewed to explore the effectiveness of digital interventions which are available for healthcare professionals who experience psychological trauma.

**Methods:** A comprehensive search was conducted across four electronic databases, Pubmed, Medline Ovid, Embase Ovid, PsychINFO Ovid, resulting in a total of six RCTs that met inclusion criteria. Assessment of study quality and risk of bias were conducted using the Jadad scale and Cochrane Risk of Bias Tool respectively. Whilst the RCTs included in the review investigated the efficacy of interventions on healthcare professionals’ wellbeing, the modalities of the interventions varied. Interventions included smartphone-based stress management modules, resilience training, smartphone applications focusing on emotional skills, cognitive-behavioural therapy exercises and psychoeducation, as well as computerised Eye Movement Desensitisation and Reprocessing (EMDR) intervention and internet intervention enhancing self-efficacy. As a result, a meta-analysis was not applicable to be carried out.

**Results:** Based on the findings, digital interventions had positive impacts on reducing the mental strain experienced by healthcare professionals. Some studies proved that the improvements were of statistical significance. The results of the RCTs in this review looked promising for the future of digital interventions targeting the mental wellbeing of healthcare professionals. In particular, the computerised EMDR intervention and the self-guided internet intervention targeting self-efficacy or social support, illustrated the potential benefits in its results. However, other studies indicated the need for further research before definitive conclusions can be drawn. The majority of the studies used a smartphone-based intervention. However, there was no correlation between the efficacy of these RCTs and this feature of their modality.

**Conclusion:** The scarce literature available in relation to this topic displayed promising evidence that digital interventions helped healthcare professionals experiencing psychological trauma.

